# A Single-Center Comparative Study: Outcome Analysis of Fixation Techniques for Tibiotalocalcaneal Arthrodesis

**DOI:** 10.7759/cureus.45308

**Published:** 2023-09-15

**Authors:** Toktamış Savaş, Burcin Karsli, Vahap Kurt, Nurcihan Yavuz Savaş

**Affiliations:** 1 Orthopaedics and Traumatology, Sanko University School of Medicine, Gaziantep, TUR; 2 Orthopeadics, Gaziantep University, Gaziantep, TUR; 3 Orthopaedics and Traumatology, Abdulkadir Yüksel State Hospital, Gaziantep, TUR; 4 Radiology, Sanko University School of Medicine, Gaziantep, TUR

**Keywords:** sf-36, aofas hind foot score, plate and screws, retrograde intramedullary nail, ankle joint arthrodesis, tibiotalocalcaneal arthrodesis

## Abstract

Introduction

Tibiotalocalcaneal arthrodesis (TTCA) is a well-known and accepted surgical technique for end-stage ankle osteoarthritis. The aim of this study is to compare the postoperative clinical and radiological comparison of retrograde intramedullary nailing (RIMN) and plate/cannulated screw (P/cS) fixation methods in patients undergoing TTCA.

Methods

Patients with end-stage ankle osteoarthritis due to traumatic causes or rheumatic diseases between December 2012 and March 2019 were included in the retrospective study. Patients who underwent isolated tibiotalar or isolated subtalar arthrodesis were not included in the study. Functional scores of patients with bone fusion were evaluated using the American Orthopaedic Foot and Ankle Society (AOFAS) and Short Form-36 (SF-36) quality of life score surveys, administered either in person or by phone. From the radiological point of view, it was evaluated whether the union was achieved with the two-view ankle radiograph. There were 48 patients who underwent ankle arthrodesis in the clinical archive. A total of 31 patients were excluded from the study due to failure to attend follow-up, inability to be reached, or non-compliance with study criteria. The mean age of the patients participating in the study was 44.12 ± 12.95 years, the follow-up time was 40.06 ± 27.31 months, the union time was 5.19 ± 3.17 months, and the AOFAS score was 53.12 ± 13.87. SF-36 scores were evaluated among their own subunits.

Results

A total of 17 patients were enrolled in the study, of whom 13 were male (76.47%) and 4 were female (23.53%). There was no significant effect of the fixation methods (RIMN or P/cS) selected for TTCA on union times (p>0.05). However, there were significant differences in some parameters of the SF-36 when compared by gender. According to this, the scores of men in physical function (PF), mental health (MH), and general health perception (GHP) were higher than those of women. When AOFAS and SF-36 scores were compared by fixation type, no statistically significant difference was found (p>0.05).

Conclusion

This study investigated the impact of the fixation method on clinical and radiological outcomes in TTCA. We found that both methods were clinically similar in terms of bone union time and surgical efficacy. However, men had better physical function, mental health, and general health perception after TTCA than women.

## Introduction

The ankle is the structure primarily responsible for the biomechanical balance between the body and the foot [1]. Despite the fact that the ankle joint complex bears a load of approximately five times the body weight during normal walking and standing and up to thirteen times the body weight during activities such as running, degenerative osteoarthritis of this structure is less common than that of the knee and hip [2]. Osteoarthritis of the ankle is a degenerative joint disease characterized by swelling, pain with weight bearing, stiffness, and decreased range of motion of the ankle joint [3]. In our study, we refer to tibiotalocalcaneal (TTC) arthritis, which is arthritis in both the subtalar and talocrural joints.

Although previous traumas are the most common factor in the etiology of ankle degenerative arthritis, deformities that disrupt the distribution load in the ankle, inflammatory arthritis (rheumatoid arthritis, systemic lupus erythematosus, or gout), and previous septic arthritis also play a role in the etiology [4,5].

Ankle and subtalar osteoarthritis, in which secondary causes are more prominent in etiology, is an important health problem in terms of patients' life comfort. Conservative treatment appears to be the first choice for solving this problem. Tibiotalar joint replacement and subtalar arthrodesis were tried on patients who did not obtain satisfactory results from conservative treatment. However, the complexity of this surgical method and its uncertain long-term results are its disadvantages [6]. TTCA showed clinically positive results in patients with tibiotalar arthritis (TTA) and subtalar arthritis (STA); it has significantly improved the quality of life of the patients and is accepted as the gold standard surgical method in patients with end-stage TTA and STA [7].

TTCA was first described by Lexer in 1906 [8]. With the development of implanted devices and surgical methods, the radiological recovery rate increased and complication rates decreased in patients who underwent TTCA. Fixation methods for TTCA: external fixator, retrograde intramedullary nailing (RIMN), and P/cS can be counted. The plate fixation method can be done with conventional or locking plates.

RIMN detection has the advantage of a fast learning curve for the surgeon. It is an effective fixation method with 71-95% fusion rates [9]. However, this method requires reaming, which may increase the possibility of infection, pulmonary embolism, and systemic inflammation [10]. Locked plate fixation in P/cS fixation, compared to conventional plate fixation, has significant advantages in the treatment of osteoporotic fractures, severe comminuted fractures, and periarticular fractures [11]. Locking plates do not have direct contact with the bone. It acts as an internal fixator. Therefore, periosteal blood supply is not inhibited by the direct pressure of the plate on the bone, as observed in dynamic compression plates (DCPs) [12,13].

Although there are many studies on implant systems used for fixation in the literature, it is still controversial which approach is better for TTCA. Thus, based on this data, we analyzed the clinical outcomes of 17 patients and intend to compare intramedullary fixation methods with other fixation methods.

## Materials and methods

The records of 17 patients who underwent TTCA with RIMN or P/cS between December 2012 and March 2019 were evaluated retrospectively. All patients diagnosed with TTA and STA underwent two-dimensional ankle radiographs. To confirm the diagnosis, computed tomography (CT) and/or magnetic resonance imaging (MRI) scans were performed. Seventeen patients were evaluated for functional scores on the American Orthopaedic Foot and Ankle Society (AOFAS) and Short Form-36 (SF-36) quality of life score questionnaires after fusion. The study was initiated after obtaining approval from the ethics committee of the authors' institution.

Operative and clinical procedure

The patients were operated on under general anesthesia or spinal anesthesia in the supine position. All surgeries were performed by the same team (TS, VK), led by an experienced ankle and foot surgeon (BK). The cartilage part was excised from the tibiotalar joints with an anteromedial or anterolateral approach. No surgical procedure was applied to the subtalar joint. A hemovac drain was used. A short leg splint was applied postoperatively. Skin sutures were removed between the 10th and 20th days after surgery. Weight-bearing was not allowed for six weeks after surgery. The patients were followed up for a maximum of six months. During this time, they were called for follow-up appointments until fusion was achieved. Upon evaluation of the ankle radiograph, the presence of callus tissue in three out of four bone cortexes of the fusion area was considered to indicate fusion. Patients with fusion were administered the AOFAS and SF-36 questionnaires.

Retrograde Intramedullary Nail Fixation Method

**Figure 1:**
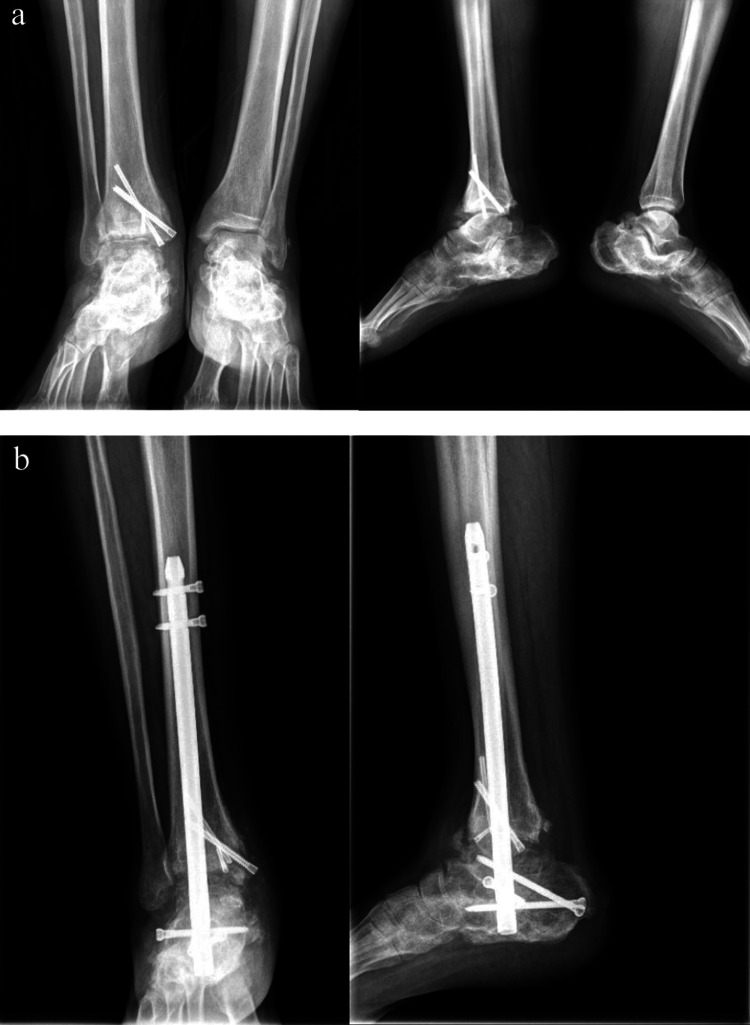
(a) Anteroposterior and lateral X-ray views of the post-traumatic patient showing tibiotalar and subtalar arthritis; (b) six months after operation X-ray views showing union by RIMN RIMN: retrograde intramedullary nailing

The entry point of the nail was determined as the junction of the long axis drawn from the second toe to the sole of the foot and the line drawn from the malleolus to the sole of the heel. The location was verified using fluoroscopy during entry. Appropriate-length nails were evaluated under fluoroscopy. The guidewire was found to be in the proper position, and all patients underwent retrograde ankle arthrodesis nail (TRIGEN Hindfoot Fusion Nail, Smith & Nephew™, TN, USA) placement (Figure 1A-1B).

Canulated Screw/Plate Fixation Method

**Figure 2:**
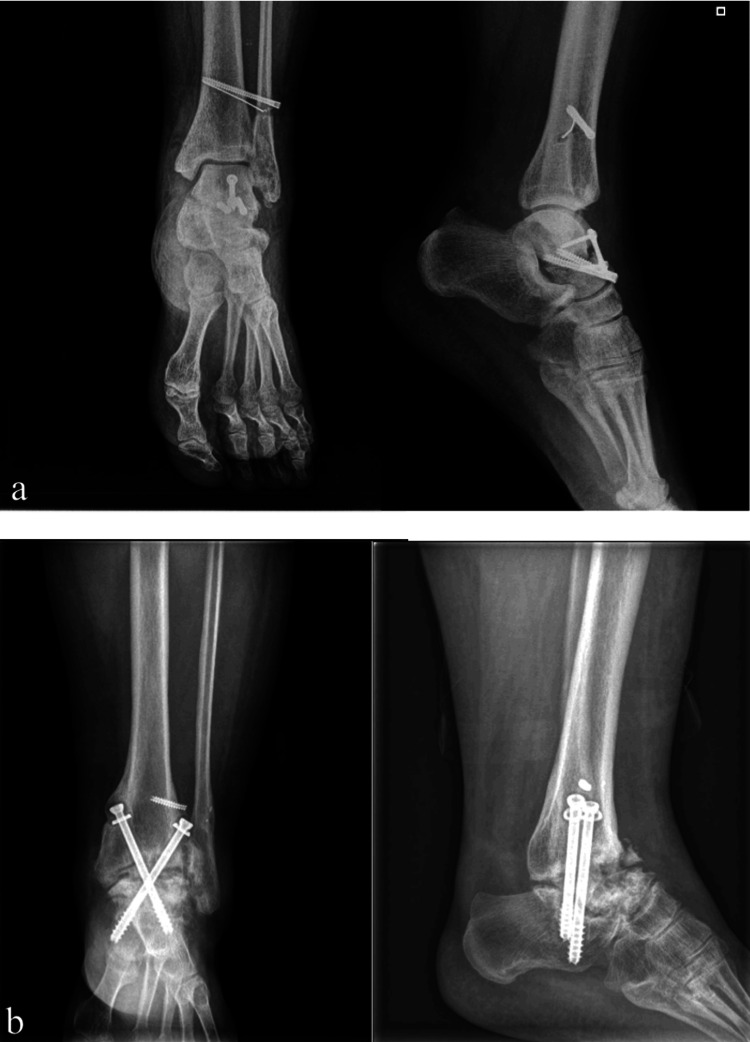
(a) Anteroposterior and lateral X-ray views of the post-traumatic patient showing tibiotalar and subtalar arthritis; (b) 6 months after operation X-ray views showing union by canulated screws

The patients were operated on with the help of a pneumatic tourniquet. The tibiotalar joint was reached with an anterior approach, and the cartilage tissue was excised. All patients underwent a locked low contact dynamic compression plate (LC-DCP) (Zimed™, Gaziantep, TR) or 7.3 mm canulated screws (Zimed™) were used under fluoroscopy control for both tibiotalar and subtalar arthrodesis fixation (Figure 2A-2B).

Stastistical analysis

The normal distribution suitability of the obtained variables was examined using the Shapiro-Wilk test. The comparison of normally distributed variables was performed using the t-test (student t-test) and the comparison of non-normally distributed variables using the Mann-Whitney U test. A value of p<0.05 was considered statistically significant. Analyses were made with the help of SPSS v22.0 (IBM Corp., Armonk, NY) for Windows.

## Results

Of the 17 patients included in the study, 13 (76.47%) were male and 4 (23.53%) were female. In total, 11 patients (64.71%) underwent intramedullary nailing and 6 patients (35.29%) underwent P/cS for fixation. Patients who met the inclusion criteria for the 2012-2019 date range were selected, regardless of the gender of the participants. No significant difference was found in terms of the fusion times of the fixation methods preferred for TTCA (4.90 ± 3.18 vs. 5.67 ± 3.39 mo, p>0.05) (Table 1). There was no significant difference in fusion times between genders (5.08 ± 2.75 vs. 5.50 ± 4.73 mo, p>0.05) (Table 2).

**Table 1:** Statistical evaluation of AOFAS and SF-36 according to fixation type AOFAS: American Orthopaedics Foot and Ankle Society, SF-36: short form-36, RIMN: retrograde intramedullary nail, P/cS: plate/canulated screw

	Fixation type	N	Mean	Standard deviation	p-Value
AOFAS Score	RIMN	11	52.55	10.46	0.826
	P/cS	6	54.17	19.86	
Physical functioning	RIMN	11	49.09	27.91	0.309
	P/cS	6	63.33	24.01	
Role limitations (physical problems)	RIMN	11	20.45	40.03	0.443
	P/cS	6	29.17	40.05	
Role limitations (emotional problems)	RIMN	11	33.36	47.16	0.142
	P/cS	6	66.67	42.22	
Vitality	RIMN	11	55.00	16.43	0.746
	P/cS	6	58.33	25.43	
Mental health	RIMN	11	58.27	20.30	0.226
	P/cS	6	66.67	23.96	
Social functioning	RIMN	11	49.00	26.43	0.171
	P/cS	6	71.00	36.55	
Pain	RIMN	11	53.27	22.10	0.868
	P/cS	6	55.50	32.55	
General health perception	RIMN	11	57.27	20.66	0.417
	P/cS	6	67.50	29.96	
Duration of bone union (month)	RIMN	11	4.90	3.18	0.655
	P/cS	6	5.67	3.39	

When AOFAS and SF-36 scores were compared according to gender, physical function (PF), mental health (MH), and general health perception (GHP) sub-parameters were statistically significant (p<0.05). As a result, males had higher scores in physical condition, general health perceptions, and mental health compared to females (62.31 ± 24.88 vs. 27.50 ± 10.41, p<0.05) (Table 2).

**Table 2:** Statistical evaluation of AOFAS and SF-36 according to gender AOFAS: American Orthopaedics Foot and Ankle Society, SF-36: short form-36, RIMN: retrograde intramedullary nail, P/cS: plate/canulated screw

	Gender	N	Mean	Standard deviation	p-Value
AOFAS Score	Male	13	55.23	11.61	0.271
	Female	4	46.25	20.12	
Physical functioning	Male	13	62.31	24.88	<0.005
	Female	4	27.50	10.41	
Role limitations (physical problems)	Male	13	26.92	43.85	0.894
	Female	4	12.50	14.43	
Role limitations (emotional problems)	Male	13	35.92	44.06	0.141
	Female	4	75.00	50.00	
Vitality	Male	13	62.31	11.66	0.153
	Female	4	36.25	27.50	
Mental health	Male	13	70.85	11.19	<0.005
	Female	4	30.00	14.79	
Social functioning	Male	13	59.77	31.02	0.492
	Female	4	47.00	34.14	
Pain	Male	13	53.77	24.41	0.935
	Female	4	55.00	31.89	
General health perception	Male	13	67.69	20.68	<0.005
	Female	4	38.75	22.13	
Duration of bone union (month)	Male	13	5.08	2.75	0.619
	Female	4	5.50	4.73	

No statistically significant difference was found when the AOFAS and SF-36 scores were compared according to the fixation type (p>0.05) (Table 1).

## Discussion

The SF-36, one of the most common generic measures used to evaluate the quality of life, is a questionnaire that examines eight dimensions of health with 36 items [14]. The result that made our study unique was the gender aspect: male patients had higher PF, MH, and GHP scores than female patients in SF-36 sub-parameters. This situation made us think about what needs to be considered in patient selection. In particular, male patients were found to have a better quality of life than female patients in terms of PF, MH, and GHP. These findings could be interpreted as suggesting that male patients may respond better to treatment or experience fewer complications. Additionally, we believe that surgeons who want to improve postoperative clinical outcomes in TTCA should be able to manage their patients' expectations well, and this requires more careful communication and counseling in female patients than in male patients.

However, further studies with a larger sample size are needed to draw definitive conclusions from our study. This is because the number of female patients in our study was only 4. To better understand the underlying causes of the difference in SF-36 scores between sexes, it is necessary to conduct large-scale studies that take into account patient demographics, medical history, and lifestyle.

The three bones that constitute the ankle-the tibia, fibula, and talus-articulate with each other. The distal tibia and the medial and lateral malleolus form the "Ankle Mortis" and envelop the talus. This complex structure makes the ankle cartilage more resistant to tensile loads and arthrosis [15]. Due to the anatomical structural protection and biomechanical properties of the chondral tissue in the ankle [16], primary osteoarthritis is more common in the hip and knee, which are larger joints of the lower extremity. Arthritis developing in the ankle is usually due to secondary causes. Because of this protected joint structure, the possibility of primary arthritis occurring in the ankle is much lower than in other regions.

Causes of ankle arthritis due to secondary causes include traumatic osteoarthritis, avascular necrosis of the talus, rheumatoid arthritis, unsuccessful total ankle arthroplasty, or neuropathic deformities [17]. TTCA, the gold standard method for treating ankle arthritis, aims to provide a stable, painless, and plantigrade foot [7]. All of the patients included in our study underwent TTCA for secondary ankle arthritis, and a stable and plantigrade foot was achieved in all our patients.

There are very few studies in the literature on the clinical outcomes of TTA and TTCA [18-20]. Of these, Ajis et al. did not find a statistically significant difference between the two surgical methods (TTA-TTCA) in their study, and they thought that the reason for this was the result of the surgeon's counseling given to the patients during the preoperative period [20]. It is known that the subtalar and chopart joints are preserved after TTA in the lower limbs. It is accepted by the authors that it can protect the biomechanical function more than TTCA [20]. The reason why we did not include patients with isolated TTA was that we thought that we would not be able to obtain accurate clinical results in our study due to both this accepted situation and the lack of studies in the literature [21].

Many methods are used for fixation in TTCA [10,22-24]. Haaker et al. and Niinimäki et al. found that RIMN had faster union times [25,26]; however, in the study of Hoppenfeld et al. [15], union with plate was faster than RIMN. Considering the results of our study, there was no significant difference between the fixation method and union time. A literature review suggests that there are several potential explanations for the observed discrepancy between the efficacy of TTC and plate fixation for ankle arthrodesis. Gavaskar and Chowdary used supracondylar femoral nails in their study, and they reported that the curved structure of the nail enabled fusion [27]. Yakacki et al. reported in their review that the third-generation TTCA nail has an elastic curve that can adapt to the curvature of the arthrodesis site, thereby achieving effective fusion [28]. We believe that the discrepancy between the results of nail and plate comparative studies is due to the variety of TTCA nails available on the market as well as the differences in bone alignment at the TTCA site. Therefore, more studies are needed to determine which method is more effective. In our study, the TTCA nail used was a first-generation TTCA nail.

In many studies in the literature, when clinical scores after TTCA were compared regarding fixation techniques, no statistical difference was found [10,24], and in our study, the effect of our fixation methods on AOFAS and SF-36 scores was not statistically significant. Postoperative clinical scoring was consistent with the literature.

Our study has some limitations, which should be noted. First, the study was retrospective. Additionally, the total number of patient samples and female patients included in the study is relatively low. Also, the fact that female genders’ PF, MH, and GHP scores were lower than males suggested that women may have fluctuations in their hormonal cycles or in the menopausal period at the time of this survey, which may lead to false results [29,30]. However, our current study may form the basis for further and more extensive clinical trials.

## Conclusions

The fixation methods used for TTCA do not have a significant impact on fusion time or clinical outcomes. However, the surgeon's experience is an important factor in the selection of the fixation method. Preoperative counseling for patients undergoing TTCA, especially for female patients, may have a positive impact on their physical function, mental health, and overall health perception.
